# Generalized Cross-Frequency Decomposition: A Method for the Extraction of Neuronal Components Coupled at Different Frequencies

**DOI:** 10.3389/fninf.2018.00072

**Published:** 2018-10-18

**Authors:** Denis Volk, Igor Dubinin, Alexandra Myasnikova, Boris Gutkin, Vadim V. Nikulin

**Affiliations:** ^1^Interdisciplinary Scientific Center J.-V. Poncelet (CNRS UMI 2615), Moscow, Russia; ^2^Institute for Cognitive Neuroscience of the National Research University Higher School of Economics, Moscow, Russia; ^3^Moscow Institute of Physics and Technology, Moscow, Russia; ^4^Group for Neural Theory, Laboratoire des Neurosciences Cognitives et Computationelles INSERM U960, Department of Cognitive Studies, Ecole Normale Superieure PSL University, Paris, France; ^5^Department of Neurology, Max Planck Institute for Human Cognitive and Brain Sciences, Leipzig, Germany; ^6^Neurophysics Group, Department of Neurology, Charité–Universittsmedizin Berlin, Berlin, Germany; ^7^Bernstein Center for Computational Neuroscience, Berlin, Germany; ^8^Center for Bioelectric Interfaces of the Institute for Cognitive Neuroscience of the National Research University Higher School of Economics, Moscow, Russia

**Keywords:** cross-frequency coupling, EEG & MEG, phase-to-phase coupling, brain oscillations, source localization

## Abstract

Perceptual, motor and cognitive processes are based on rich interactions between remote regions in the human brain. Such interactions can be carried out through phase synchronization of oscillatory signals. Neuronal synchronization has been primarily studied within the same frequency range, e.g., within alpha or beta frequency bands. Yet, recent research shows that neuronal populations can also demonstrate phase synchronization between different frequency ranges. An extraction of such cross-frequency interactions in EEG/MEG recordings remains, however, methodologically challenging. Here we present a new method for the robust extraction of cross-frequency phase-to-phase synchronized components. Generalized Cross-Frequency Decomposition (GCFD) reconstructs the time courses of synchronized neuronal components, their spatial filters and patterns. Our method extends the previous state of the art, Cross-Frequency Decomposition (CFD), to the whole range of frequencies: it works for any *f*_1_ and *f*_2_ whenever *f*_1_:*f*_2_ is a rational number. GCFD gives a compact description of non-linearly interacting neuronal sources on the basis of their cross-frequency phase coupling. We successfully validated the new method in simulations and tested it with real EEG recordings including resting state data and steady state visually evoked potentials (SSVEP).

## 1. Introduction

Synchronization between neuronal populations is considered to be a key mechanism underlying interactions between distinct groups of neurons. According to the communication-through-coherence (CTC) hypothesis, efficient communication between two groups of neurons is only possible, when the oscillations are phase-locked (coherent) (Fries, 2015).

Among the various synchronization phenomena, interactions within the same frequency band (with ratio 1:1, i.e., gamma-gamma, alpha-alpha or beta-beta) have been mostly studied and well characterized both in humans (Varela et al., 2001; Halgren et al., 2002; Howard et al., 2003; Palva et al., 2005) and in animals (Womelsdorf et al., 2017). These coherent interactions are present both during tasks and during the resting state. In an MEG study it has been shown that pronounced 1:1 phase synchrony is present in resting state for all frequency bands and over the whole cortex whereas local 1:*r*, *r* ≥ 2 , phase synchrony is increased during problem solving in the beta-alpha and gamma-alpha range over the right hemispheric posterior regions (Palva et al., 2005). Meanwhile, long-range alpha-band phase synchronization is associated with attentional and working memory processing (Palva and Palva, 2011). Synchronization in beta and gamma frequency ranges and their spatial patterns indicate the activation of visual attention and forming of visual representation (Siebenhühner et al., 2016). It has been established that beta phase synchronization accompanies auditory-motor rhythm learning (Edagawa and Kawasaki, 2017). Gamma range zero phase synchronization can be associated with sensory representation in the olfactory bulb (Li and Cleland, 2017).

In addition to within frequency synchronization, coupling between two frequency bands has also been observed in humans (Sauseng et al., 2008; Jirsa and Müller, 2013; Akiyama et al., 2017; Palva and Palva, 2017) and animal recordings (Chrobak and Buzsáki, 1998). Such cross-frequency synchronization of neuronal oscillations in two distinct frequency bands is an active topic of investigation (Palva and Palva, 2017). In fact, recent experimental results suggest that cross-frequency coupling between spatially distributed sources may underlie dynamic formation of functional brain networks involved in perceptual, cognitive and motor performance (as reviewed in Hyafil et al., 2015).

Several types of cross-frequency coupling have been revealed: amplitude-to-amplitude, phase-to-phase, amplitude-to-phase cross-frequency coupling (Canolty et al., 2006; Jensen and Colgin, 2007). According to the recent findings, different cross-frequency coupling types are associated with various functional roles. For example, theta oscillations affect phase or power of gamma oscillations in the auditory circuits during speech processing (Giraud and Poeppel, 2012; Hyafil et al., 2015). Cross-frequency coupling between gamma power and alpha phase reflect individual ability to encode memory (Park et al., 2016).

A particularly important form of neuronal interactions is phase-to-phase synchronization since it represents stable spike-time relationships between distant neuronal oscillations and, therefore, it directly coordinates phase coupling of fast and slow oscillations (Siebenhühner et al., 2016). Phase-to-phase synchronization is believed to integrate and coordinate neuronal activity (Jirsa and Müller, 2013; Akiyama et al., 2017). There is evidence that cross-frequency phase synchrony between theta and alpha-gamma and between alpha and beta-gamma oscillations reflect the load in working memory tasks (Siebenhühner et al., 2016). Cross-frequency phase coupling underlies recent animal neurophysiological observations: theta-gamma neuronal interactions in the hippocampus (Belluscio et al., 2012). Interactions between alpha, theta, beta and gamma band oscillations in fronto-cortical areas and its modulation appear to play a crucial role in higher cognitive functions (Palva and Palva, 2017), such as working memory (Chaieb et al., 2015), memory integration and attentional processes (Sauseng et al., 2008).

Albeit potentially important for cognitive function and brain computation, phase-to-phase coupling is not easy to characterize in noninvasive recordings, since the prevalent methods suffer from a number of difficulties, such as non-sinusoidal nature of oscillations, non-stationarity of the signals and large amount of noise in EEG data (Nikulin and Brismar, 2006; Hyafil et al., 2015; Lozano-Soldevilla et al., 2016). A particularly difficult problem relates to volume conduction, which leads to the simultaneous detection of the same signal at many sensors thus complicating the extraction of the individual neuronal sources showing cross-frequency synchronization. One way to solve this problem is to use inverse source modeling. This approach, however, also has limitations due to non-uniqueness of the obtained solutions. Finally, spatial decomposition techniques might be used to extract coupled signals, such as SPoC (Source Power Comodulation) and CFD (Cross Frequency Decomposition). The latter, while being highly efficient, is limited to 1:*r* ratio (Nikulin et al., 2012). Hence, new techniques for reliable estimation of cross-frequency phase coupling are needed.

In order to avoid the limitations of the previous methods, in the present study we propose a new approach for the extraction of components demonstrating cross-frequency phase coupling. We refer to this new method as Generalized Cross-Frequency Decomposition (GCFD). The GCFD is a generalization of CFD (Nikulin et al., 2012) and features non-linear techniques to extract the strongest rhythmic components coupled at *p*:*q* frequency ratio. The method is applicable to a wide range of cross frequency interactions compared to previous state-of-art CFD. We tested the performance of the GCFD both in simulations and on real EEG data (steady state visually evoked potentials (SSVEP) and resting state datasets).

## 2. Methods

### 2.1. Cross-frequency phase synchrony

Let *s*(*t*) be the time course of a real-valued signal. The instantaneous phase of *s*(*t*) is defined as follows. The signal *s*(*t*) is first complexified:

$$\begin{array}{l} \tilde{s}\left(t\right)=s\left(t\right)+iH\left(s\right)\left(t\right), \end{array}$$

where *H*(·) is Hilbert transform and *i* is the imaginary unit. Complex-valued signal $\tilde{s}\left(t\right)$ is called *analytic signal* for *s*(*t*) and admits the following factorization

$$\tilde{s}\left(t\right)=A\left(t\right)\cdot e^{i\varphi \left(t\right)},\;A\left(t\right)\in \mathbb{R},\;A\geq 0,\;\varphi \left(t\right)\in \mathbb{R},$$

where *A*(*t*) is called *instantaneous amplitude* of *s*(*t*), and φ(*t*), defined modulo 2π, is called its *instantaneous phase*.

To study ordinary 1:1 phase synchrony of two narrow-band signals *s*_1_(*t*) and *s*_2_(*t*) with the same central frequency *f*, we observe their cyclic phase difference

$$\Delta \varphi \left(t\right):=\varphi_{2}\left(t\right)-\varphi_{1}\left(t\right)\;\mathrm{mod}\;2\pi$$

in a long enough time window *t* = 1..*T*. In the case of total absence of synchrony, the empirical probability distribution of Δφ(*t*) across the time window would be uniform on the segment [0, 2π]. Any significant deviation from the uniform distribution indicates presence of an interaction between the instantaneous phases of *s*_1_(*t*) and *s*_2_(*t*). A strong form of this is when Δφ(*t*) has a strongly pronounced unimodal distribution on [0, 2π]. This means that for some *c*∈[0, 2π], at almost every time moment we observe that φ_2_(*t*)−φ_1_(*t*)≈*c*. This is called *phase locking*.

Now let us generalize this to the case when the central frequencies *f*_1_ and *f*_2_ of *s*_1_(*t*) and *s*_2_(*t*) are different but rationally related:

$$f_{1}:f_{2}=p:q,\;p,q\in \mathbb{N},$$

see Rosenblum et al. (2001). Study of synchrony between such signals is the primary goal of this paper. To analyze this case, we again calculate their instantaneous phases φ_1_ and φ_2_ but now we consider generalized cyclic phase difference

$$\Delta \varphi_{p,q}\left(t\right):=p\varphi_{2}\left(t\right)-q\varphi_{1}\left(t\right)\;\mathrm{mod}\;2\pi .$$

Now we say that *s*_1_ and *s*_2_ are in *cross-frequency phase synchrony (CFS)* if the distribution of Δφ_*p, q*_(*t*) is non-uniform.

We use the following *Phase Locking Value* (Rosenblum et al., 2001; Palva et al., 2005) to estimate the strength of CFS between *s*_1_ and *s*_2_ in the time window *t* = 1..*T*:

(1) $$\begin{array}{r} PLV_{p,q}\left(\varphi_{1},\varphi_{2}\right)=\frac{1}{T}|\underset{t=1..T}{\sum }e^{i\Delta \varphi_{p,q}\left(t\right)}|. \end{array}$$

### 2.2. Preprocessing

In case of empirically obtained recordings, raw E/MEG data are inspected and cleaned of blinking and other muscle artifacts. This is done by applying Independent Component Analysis (FastICA algorithm, see Hyvärinen et al., 2001) and removing all the artifact-related components. In the end, we have multi-channel E/MEG signal with one or more continuous time intervals clear of artifacts, called *epochs*.

Cleaned E/MEG data is then low-pass filtered into a wide band starting from 0.5 Hz to about 150% of the highest frequency to be used in the analysis. If we are interested in cross-frequency interaction between a 20 Hz rhythm and a 30 Hz rhythm, the high cut-off frequency of the filter would be 45 Hz. This is done to further clean the data of any high-frequency noise.

### 2.3. The GCFD algorithm

**Figure 1:**
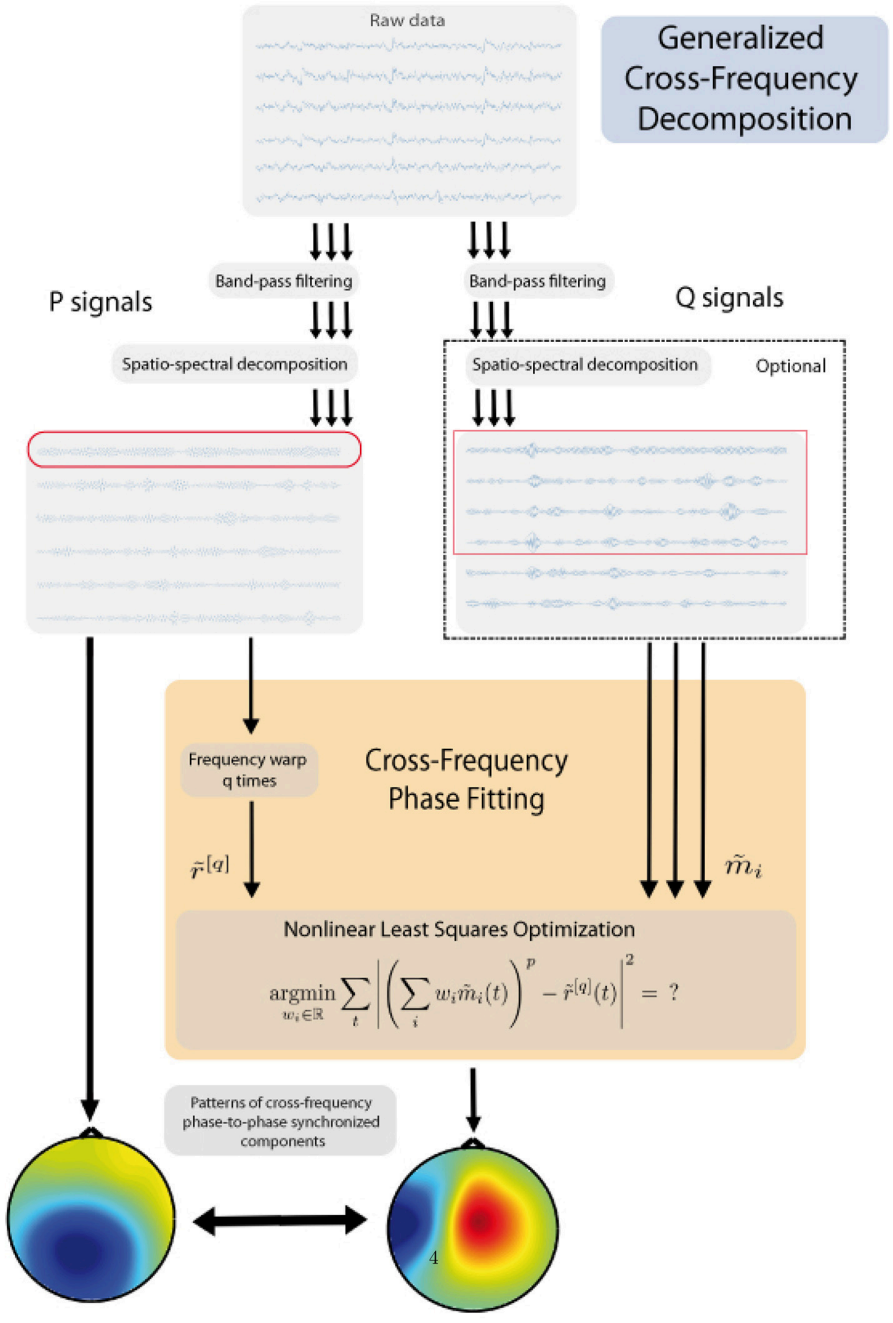
Outline of the algorithm.

The general workflow of the proposed method is presented in Figure 1 and details will be presented in the subsequent sections. The method consists of the following principal steps:

- choose one band that represents a “*reference”* band - P, while another represents a “*fit”* band - Q;
- identify one or few candidate components for the reference band;
- for the other frequency, using non-linear optimization find the unique components which are in the strongest synchrony with the reference rhythm candidates;
- output the pair(s) which exhibit the best synchronization.

Below we address each of these steps in detail, as well as some auxiliary steps.

#### 2.3.1. Reference band choice

In the following analysis, the frequency bands *f*_1_ and *f*_2_ play two different roles. First, we pick a few strongest rhythmic components from the reference band using Spatio-Spectral Decomposition, see Subsection 2.3.2. These components become our reference signals. Second, for each of the reference signals, we find the component in the fit band which is the most synchronous to the rhythmic activity in the reference signal. This is done with the new Cross-Frequency Phase Fitting (XPF) method which is the core of this paper.

When there is no particular reason to prefer *f*_1_ over *f*_2_ or vice versa as a reference band, we recommend choosing the band with smaller frequency. This way polynomial expressions in the nonlinear optimization procedure have lower degrees and thus the method converges faster and is more accurate. In the following, we always assume that *f*_1_ is the reference band and *f*_2_ is the fit band.

We emphasize here that XPF is asymmetric with respect to the order of the frequency bands *f*_1_ and *f*_2_. That is, the same analysis but with the bands swapped places is not, in general, guaranteed to yield the same results. However numerical experiments on simulated data indicate that, while the results might be different, both approaches are very close to the ground truth. Spatial patterns for the components in the fit band are, on average, found more accurately then spatial patterns for the components in the reference band, we elaborate on this in the sections below.

#### 2.3.2. Spatio-spectral decomposition

For the reference frequency band *f*_1_, we perform a decomposition procedure which allows us to extract relevant oscillatory components and to reduce the dimensionality of the data. Among such procedures, Spatio-Spectral Decomposition (SSD) method (Nikulin et al., 2011) showed particularly good results in simulation tests. This is because SSD is better tailored to treat narrow-band signals as compared to more general methods such as ICA.

Essentially, SSD maximizes the Signal-to-Noise Ratio which is defined as the ratio of the power at the narrow frequency band of interest to the power of the noise at the surrounding flanking frequency ranges. See Nikulin et al. (2011), for the full description of SSD and (Nikulin et al., 2012), section 3 for its compact outline. We employ SSD in GCFD two times. First time we use SSD to extract the strongest rhythmic components in the reference band, see Subsection 2.3.1. Second time we perform SSD to reduce computational complexity of the nonlinear optimization problem by lowering the effective number of components in the fit band, see Subsection 2.4.

For the following analysis, from the reference band we take only the components with the largest eigenvalues, and discard the rest of the reference band signal space. Each particular dataset may have different numbers of these significant components. We recommend first using the default number of 5 components, and adjust it later if needed.

#### 2.3.3. Cross-frequency phase fitting (XPF)

Cross-Frequency Phase Fitting (XPF) is the core procedure of GCFD. Its inputs are a single narrow-band reference signal *r*(*t*) at a frequency *f*_1_ and a collection of multiple narrow-band signals *m*_1_(*t*), …, *m*_*I*_(*t*) at a frequency *f*_2_, where *f*_1_:*f*_2_ = *p*:*q* with positive integers *p, q*, and *t* = 1..*T*. During the standard GCFD workflow, the reference signals are components from SSD which are fed into XPF one by one. Another possible scenario described in Subsection 2.5 is when the single reference signal is known *a priori*. Then XPF is applied only once using this particular signal and a set of signals in the fit band.

We assume that *m*_*i*_ is a linear combination of an unknown “target” signal *s*(*t*) which is in cross-frequency phase synchrony (CFS) with *r*(*t*), and *I*_*N*_ noise components *n*_*i*_:

(2) $$\begin{array}{r} {\left(\begin{array}{c} m_{1}\\ \vdots \\ m_{I} \end{array}\right)}^{T}={\left(\begin{array}{c} s\\ n_{1}\\ \vdots \\ n_{I_{N}} \end{array}\right)}^{T}\cdot P, \end{array}$$

where *P* is a real-valued (*I*_*N*_ + 1)-by-*I* matrix called *spatial mixing matrix*, or *spatial pattern matrix*. We first aim to recover the coefficients *w*_*i*_ ∈ ℝ such that $s\left(t\right)=\underset{i}{\sum }w_{i}m_{i}\left(t\right)$. These coefficients are called *spatial filter*, or simply *filter*. If *P* was a square matrix, then *w*_*i*_ would be the first column of *P*^−1^.

For every channel *r*(*t*) and *m*_*i*_(*t*) we compute their complex analytic signals $\tilde{r}\left(t\right)=r\left(t\right)+iH\left(r\right)\left(t\right)$ and ${\tilde{m}}_{i}\left(t\right)=m_{i}\left(t\right)+iH\left(m_{i}\right)\left(t\right)$, where *H*(·) is Hilbert transform and *i* is the imaginary unit. Signals $\tilde{r}\left(t\right),{\tilde{m}}_{i}\left(t\right)$ are ℂ-valued time series such that for every *t* = 1..*T* holds

$$\text{Re}\left(\tilde{r}\left(t\right)\right)=r\left(t\right),\;\text{Re}\left({\tilde{m}}_{i}\left(t\right)\right)=m_{i}\left(t\right).$$

Now for $\tilde{r}\left(t\right)$ we perform so-called *frequency warp by q times*. Namely, we make up a new ℂ-valued signal $\tilde{r}\left[q\right]\left(t\right)$ such that

(3) $$\begin{array}{r} |\tilde{r}\left[q\right]\left(t\right)|=|\tilde{r}\left(t\right)|,\;\text{Arg}\left(\tilde{r}\left[q\right]\left(t\right)\right)=q\cdot \text{Arg}\left(\tilde{r}\left(t\right)\right). \end{array}$$

An equivalent way to write this down is

$$\tilde{r}\left[q\right]\left(t\right)=\frac{\tilde{r}{\left(t\right)}^{q}}{|\tilde{r}\left(t\right){|}^{q-1}}\text{for each}t\in 1..T.$$

Note the difference between the notations (·)^[*q*]^ and (·)^*q*^. We keep the latter for the standard complex power

$$z^{q}=\underset{q\text{times}}{\underset{︸}{z\cdot \ldots \cdot z}}.$$

By construction, whenever some other signal *g*(*t*) displays a *p*:*q* synchronization with $\tilde{r}\left(t\right)$, it is also in *p*:1 synchronization with $\tilde{r}\left[q\right]\left(t\right)$, and vice versa. The *q*-th power $\tilde{r}{\left(t\right)}^{q}$ of the signal $\tilde{r}\left(t\right)$ has the same property but it is less convenient for computational purposes because its magnitude $|\tilde{r}\left(t\right){|}^{q}$ could either be very small or very large for large values of *q*.

As indicated in Nikulin et al. (2012), maximization of correlation between narrow-band signals is similar to maximization of their coherence. It has been observed earlier that coherence primarily reflects phase synchronization (Nolte et al., 2004), yet it also measures amplitude correlation between the two signals. Coherence represents phase synchronization weighted by the amplitude co-modulation (Nolte et al., 2004; Friston et al., 2012). Mezeiová and Paluš (2012), however, showed that in practice phase synchronization (as measured through synchronization index) and coherence might give similar results. In addition, Nolte et al. (2004) observed that for empirical signals, it is not entirely clear whether one can assume independence between the amplitude and phase. Moreover, the authors argued that for very low signal-to-noise ratio the phase can be strongly affected and thus coherence (which includes amplitude covariation) can give more robust results than the synchronization index.

Based on this evidence, we will be maximizing the correlation between ℂ-valued signals ${\tilde{s}}_{1}^{p}$ and ${\tilde{s}}_{2}$, where ${\tilde{s}}_{1}=s+iH\left(s_{1}\right)$, ${\tilde{s}}_{2}=s+iH\left(s_{2}\right)$ are the analytic signals for *s*_1_, *s*_2_. We can find the coefficients *w*_*i*_ as the solution to the optimization problem

(4) $$\begin{array}{r} \underset{w_{i}\in \mathbb{R}}{\text{argmin}}\underset{t}{\sum }|{\left(\underset{i}{\sum }w_{i}{\tilde{m}}_{i}\left(t\right)\right)}^{p}-\tilde{r}\left[q\right]\left(t\right){|}^{2}=? \end{array}$$

Note that, while both ${\tilde{m}}_{i}\left(t\right)$ and $\tilde{r}\left[q\right]\left(t\right)$ are complex-valued, we are still looking for the coefficients *w*_*i*_ in the *real* space. Thus, this is a particular case of a Constrained Nonlinear Least Squares Problem (see, for instance, Schittkowski, 1988). A standard approach is to start from a random guess for *w*_*i*_ and iteratively descend to the local minimum. Practically, multiple modern high-level computation suites offer compact solvers for Constrained Nonlinear Least Squares Problems. In our implementation we used *lsqnonlin* function from MATLAB. Those who prefer Python can use *scipy.optimize.least_squares* function from SciPy package which provides a similar functionality.

#### 2.3.4. Back to spatial patterns

Finally, for each reference component *r*(*t*) in the frequency band *f*_1_, we have the coefficients of the spatial filter *w*_*i*_ and the component *s*(*t*) = ∑*w*_*i*_*m*_*i*_(*t*) in the fit frequency band *f*_2_ which is in cross-frequency phase synchrony with *r*(*t*). Now we explicitly compute the cross-frequency Phase-Locking Value (1) of *r*(*t*) and *s*(*t*) and choose the pair(s) (*r, s*) with the highest PLV.

The last step is to convert the spatial filters for the newly found components into the corresponding spatial patterns (see Haufe et al., 2014). An approach proposed by Parra et al. (2005) is based on assumption that the sources are mutually uncorrelated, and yields a compact formula

$$p=\frac{M^{T}s}{s^{T}s},$$

where *p* is the sought pattern, $s\left(t\right)=\underset{i}{\sum }w_{i}m_{i}\left(t\right)$, and *M* = (*m*_*i*_) is the matrix of sensor space signals in *f*_2_ frequency band. This calculation is equivalent (Haufe et al., 2014) to the multiplication of the covariance matrix of the data in *M* with the filter *w*.

The final output of the algorithm is the most synchronous pair (*r, s*) along with the respective spatial filters and spatial patterns. These spatial patterns can now be visualized as scalp topographies, see example on **Figure 3**.

### 2.4. Secondary SSD

Because the computational complexity of the core nonlinear optimization problem rapidly increases as the number of channels *N* grows (approximately, by order of ~*N*^3^, depending on a particular solver), it is sometimes beneficial to reduce the dimension of the problem. For this we project the original sensor space into a linear subspace of fewer dimensions. We apply Spatio-Spectral Decomposition (see Subsection 2.3.2) to the narrow-band signals at frequency *f*_2_ and neglect all but 15 most significant components. For example, for *N* = 60 sensors this yields a speed-up of approximately 4^3^ = 64 times. For a larger number of sensors, this step becomes even more critical.

Then we apply the same nonlinear optimization procedure (see Subsection 2.3.3) to the same reference signal and the first 15 SSD components as fit signals. This is possible because the optimization actually employs no information about the real nature of sensors' positions and works equally well for such “virtual sensors.” Finally, each spatial pattern *p*′ of length 15 found in the space of virtual sensors has to be converted back to the original sensor space:

$$p=S\cdot p^{\prime },$$

where *p* is a spatial pattern of length *N* in the space of real sensors and *S* is a *N* × 15 matrix of SSD components' spatial patterns.

Note that this speed optimization might come at a cost of reduced accuracy of the algorithm, because the search for the best spatial filter is then performed in some 15-dimensional subspace rather than the whole filter space. Thus for some cases one may consider to disable this option. However, this option is extremely useful for a fast rough search for synchronized components across many frequency bands' combinations.

### 2.5. Reference signal *r* known *a priori*

In some experimental scenarios, we may want to study synchronization of brain oscillations to a certain signal which is already known. For example, Bayraktaroglu et al. (2011) conducted a study of cortico-muscular coherence between a single-channel electromyogram signal and a multi-channel EEG data. Another example is the entrainment of neuronal activity in visual cortex to a periodic screen flickering. In such a case, an SSD step is not required prior to the main nonlinear optimization procedure. Another relevant example for this scenario is a local field potential (LFP) recording with good SNR. In all these cases the method skips the steps (a) and (b), recall Subsection 2.3, and proceeds straight to (c) since we need just the XPF procedure without the rest of the GCFD algorithm. We will call this truncated version of the workflow the *detached XPF procedure*.

We conducted numerical simulations to assess the capability of detached XPF to tackle these situations with the known reference signals. These tests showed that in this mode the overall pattern reconstruction quality for synchronized sources is significantly better than of a more sophisticated full GCFD algorithm (see below). Moreover, the detached XPF is capable of precise source reconstruction at greater values of *p* and *q*, tested up to *p, q* = 5. See Subsection 3.1 for details.

### 2.6. General phase locking

In the above, we searched for components in cross-frequency phase synchrony:

$$p\varphi_{2}-q\varphi_{1}\;\mathrm{mod}\;2\pi \approx 0.$$

A weaker condition called *cross-frequency phase locking* requires only a constant difference between the adapted cyclic phases of the signals:

$$p\varphi_{2}-q\varphi_{1}\;\mathrm{mod}\;2\pi \approx const.$$

To search for cross-frequency phase-locked components in a E/MEG signal, we modify the formula (3) for the frequency warp of reference components. We choose an integer *K*≈10 and define a complex-valued signals $\tilde{r}\left[q,k\right]$ as

(5) $$\begin{array}{r} |\tilde{r}\left[q,k\right]\left(t\right)|=|\tilde{r}\left(t\right)|,\;\text{Arg}\left(\tilde{r}\left[q,k\right]\left(t\right)\right)=q\cdot \text{Arg}\left(\tilde{r}\left(t\right)\right)+\frac{k}{K}2\pi \end{array}$$

for *k* = 0..*K*−1. Then we run the above algorithms for each *k* = 1..*K*−1, and look for the components with the highest PLV among all the runs.

Our preliminary analysis has shown that the distribution of phase differences between components is quite broad thus indicating that there is no need to exactly align the phases of both signals to have 0 or π difference. A similar number of *K* = 12 has been successfully used in another study for coherence optimization (Bayraktaroglu et al., 2012).

### 2.7. Simulations

For algorithm performance tests we picked *p*:*q* ratios which are most likely to demonstrate cross-frequency phase synchrony in human brain E/MEG recordings:

(6) $$\begin{array}{r} p:q=1:2,1:3,1:4,2:1,2:3,3:1,3:2,3:4,4:1,4:3. \end{array}$$

We aimed at simulating phase couplings between theta, alpha, beta and low gamma oscillations. Some of them, like alpha:beta (1:2), were previously observed in E/MEG recordings. For each ratio *p*:*q* we ran 100 independent randomized simulations of brain activity and tested how accurate was our algorithm reconstructing the true synchronized sources.

For each simulation we first generated 5 independent pairs of cross-frequency synchronized oscillatory signals with different frequency ratio *p*:*q*. The procedure for each pair was as follows. We generated 150 seconds length of white noise, sampled at 200 Hz frequency. This noise was then band-pass filtered in 9-11 Hz frequency range using two passes of Butterworth filter, one forward and one backward. Such two passes allow for canceling out any phase distortion caused by artifacts of a single pass of a filter (Mitra and Kuo, 2001). Then we frequency warped two copies of this signal by *p* and by *q* times, respectively. This provided us with a pair of *p*:*q*-synchronized signals in frequency bands around 10*p* Hz and 10*q* Hz.

In addition we used 100 mutually independent noise sources with 1/*f* power spectrum (so-called *pink noise*). Such a power spectrum is typical for E/MEG human brain recordings.

Each of 10 synchronized oscillatory signals and 100 noise signals coresponded to a respective current dipole randomly chosen from the nodes of triangularly tesselated cortical mantle. Dipole orientation was also randomized. We used a realistic three compartment volume conductor forward model (Nolte and Dassios, 2005) based on the Montreal Neurological Institute (MNI) head (Evans et al., 1994) to calculate the simulated EEG sensor signals from the source signals. Each simulated EEG recording had 64 channels corresponding to the standard sensor positions.

In addition, we normalized the signal-to-noise ratios (SNR) of all our signals, which we define as the ratio of the mean variance of signals across channels for each projected signal dipole and the mean variance across channels for the whole projected noise cumulative. We tested the performance of our algorithm for SNR values of 0.1, 0.5, 1.0, 2.0, see below.

Then for each simulation we ran the source reconstruction GCFD algorithm explained in Subsections 2.2–2.4. The resulting spatial patterns for the recovered sources in *f*_1_ frequency band are then compared to the true patterns which are known *a priori* by design.

In addition to these simulations we have also produced simulations for 2:3 case (SNR=0.5) mixing five components at 20 Hz frequency range but the corresponding 30 Hz components were produced by the frequency warping of another five 10 Hz components which were independent of the first 10 Hz components. This way we produced five components at 20 and 30 Hz frequency bands which were independent within and between these frequency bands. Then we performed 100 of such simulations and calculated the mean pattern divergence (see next section).

### 2.8. Pattern divergence estimation

#### 2.8.1. Simulated data

We measured the difference ϵ between the true pattern $\hat{p}$ and the reconstructed pattern *p* using the following *pattern divergence number* based on correlation between $p,\hat{p}$:

(7) $$\begin{array}{r} \epsilon =1-\frac{\left|p^{T}\hat{p}\right|}{\left||p||\cdot ||\hat{p}|\right|},\;0\leq \epsilon \leq 1. \end{array}$$

see Nikulin et al. (2012), Equation (20). The value ϵ = 0 corresponds to perfectly collinear *p* and $\hat{p}$ while ϵ = 1 stands for orthogonal *p* and $\hat{p}$.

Note that the recovered patterns come in no specific order related to the original patterns. A sorting procedure is required to find the actual recovery error for each signal. Namely, we calculated all the pairwise pattern divergences between all the recovered patterns and all the original patterns. Then we used a greedy algorithm to match the recovered patterns with the original patterns: we first find the pair of a recovered pattern and an original pattern with the smallest divergence, and then we remove both of them from the pattern sets. Then we repeat the procedure with the remaining patterns to find the second best match *et cetera*.

Each simulation yields a vector of 5 pattern divergence numbers. Multiplied by 100 simulations, in the end we have 500 numbers for each frequency ratio *p*:*q* and each SNR value. See Subsection 3.2 and **Figure 3** for results and discussion.

#### 2.8.2. Empirical data

For the empirical data, we lack the information about the ground truth patterns, and thus we cannot directly measure the divergences between the ground truth patterns and the estimated patterns. In this case we rather use pattern divergence as a measure of similarity between the two patterns relating to cross-frequency coupled components.

### 2.9. Real EEG recordings

#### 2.9.1. Resting state

The GCFD algorithm has been tested on EEG data obtained at the Centre for Cognition and Decision Making at Higher School of Economics (HSE, Moscow). All the experimental procedures were approved by the local Ethics Committee. The participants signed an informed consent form. 32 healthy subjects (12 men, right-handed, mean age 23 years) participated in the EEG experiment. The EEG data were recorded with 60 active electrodes of BrainVision actiCHamp (Brain Products GmbH) according to the extended version of the 10–20 system. The data were sampled at 500 Hz. Active channels were referenced against the mean of two mastoid electrodes. The electrooculogram was recorded with electrodes placed at the outer canthi and below the right eye. The EEG recordings were offline filtered in the frequency range 0.5–40 Hz. Spectral analysis by means of FFT (fast Fourier transform) was performed with Hammings̀ window of 3 seconds. Participants were seated comfortably before a dark screen for 10 minutes while fixating their eyes on the cross in front of them.

For the consecutive offline analysis the EEG data were downsampled to 200 Hz, the data length was 10 minutes. We reduced the dimension of the signal using the 5 strongest SSD components in both frequency ranges of interest p^*^f_1_ ± 1 Hz and q^*^f_2_ ± 1 Hz. The settings for SSD were as follows: cut-off frequency range for the band-pass filter was p^*^f_1_ ±1 Hz and q^*^f_2_ ± 1 Hz; cut-off frequency range for the lowest and highest frequencies defining flanking intervals was p^*^f_1_ ± 3 Hz and q^*^f_2_ ± 3 Hz; cut-off frequency range for the band-stop filter was p^*^f_1_ ± 2 Hz and q^*^f_2_ ± 2 Hz. We looked for the strongest synchronous components for *p*:*q* equal to 2:1 and 2:3 and and the base frequencies from the alpha frequency range (8–12 Hz). For example, the maximum PLV for *p*:*q* = 2:3 could have been attained at the base frequency 9.5 Hz which would mean that the most 2:3-synchronized components are at 19 Hz and 28.5 Hz.

#### 2.9.2. Steady state visual evoked potentials

To demonstrate performance of the GCFD we used EEG data obtained at the Centre for Cognition and Decision Making at Higher School of Economics (HSE, Moscow) with Steady State Visually Evoked Potentials (SSVEP), which were recorded for BCI experiments (Işcan and Nikulin, 2018). All the experimental procedures were approved by the local Ethics Committee. The participants signed informed consent form. 24 healthy subjects (age span 18–41 years) took part in the experiment. EEG was recorded with the sampling frequency 1 kHz with ActiCHamp amplifier using PyCorder software (Brain Products) from 60 channels actiCHamp. The band-pass filter with cut-off frequencies of 0.53 and 40 Hz was applied to raw data to remove DC component and high frequency artifacts. During the experiment the subjects were required to look at a computer screen with a single periodically flickering circle. This setup is known to evoke periodic potentials, known as Steady State Visual Evoked Potentials (SSVEPs) in subjects' visual cortex at the same frequency as the flickering frequency (Friman et al., 2007). The experiment was split into 3-second segments. For each segment the flickering frequency was randomly chosen among four fixed values: 5.45, 8.57, 12, 15 Hz.

For the consecutive GCFD analysis we concatenated all 3-second trial recordings into a single 75-second multi-channel signal for each subject and each flickering frequency. Since the raw data was filtered with high-pass at 0.53 Hz before the concatenation, there were no offsets between the epochs and thus the following filtering did not produce artifacts as was also confirmed by the visual inspection.

We reduced the dimension of the signal using the 15 strongest SSD components in both frequency ranges of interest p^*^f_1_ ± 1Hz and q^*^f_2_ ± 1Hz. For SSD we used following settings: cut-off frequency range for the band-pass filter was p^*^f_1_ ± 1Hz and q^*^f_2_ ± 1Hz; cut-off frequency range for the lowest and highest frequencies defining flanking intervals was p^*^f_1_ ± 3Hz and q^*^f_2_ ± 3Hz; cut-off frequency range for the band-stop filter was p^*^f_1_ ± 2Hz and q^*^f_2_ ± 2Hz. For computational convenience in our analysis we approximated the real flickering frequencies 5.45, 8.57, 12, 15 Hz with the integer frequencies 6, 9, 12, 15 Hz respectively.

#### 2.9.3. Statistical testing

We used the nonparametric permutation test to evaluate statistical significance of the results (Maris and Oostenveld, 2007). In our approach a test statistic was obtained from GCFD applied to randomly permuted data.

We divided recordings and combined segments in random order from the data relating to finding a spatial filter *w* while preserving the order of the segments in the reference signal which is described in section 2.3.1. Note that this randomization has been performed before finding spatial filters *w* so that all other steps are preserved like in the main analysis.

Next, we ran our algorithm on the permuted recording and obtained new paired signal for the reference signal. Then we created permutation distribution by repeating this procedure 1000 times and computing for each pair a corresponding phase locking value (1). The null hypothesis under this permutation test was that all permuted pairs and original pair belonged to the same distribution. Finally we computed the P-value for original pair of signals and if it was smaller than 0.05 we concluded that the result was statistically significant.

This is a frequently used approach for non-parametric permutation testing (Hesterberg et al., 2005; Maris et al., 2007) which preserves the spectra of the signals and all the optimization steps thus representing a robust procedure for controlling effects of the overfitting.

## 3. Results

### 3.1. Detached XPF test

**Figure 2:**
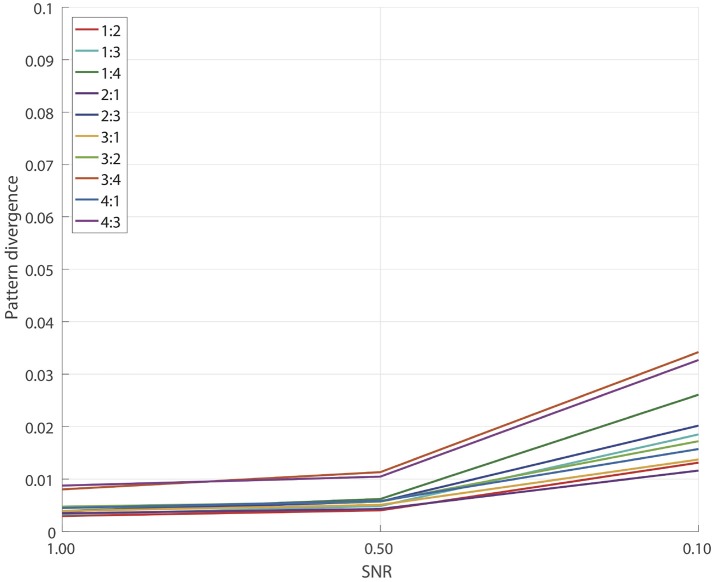
Pattern reconstruction accuracy of detached XPF.

First we tested how accurately the detached XPF procedure (recall Subsection 2.3.3) recovers source spatial patterns in the scenario when true sources are provided as reference signals and thus we only have to find cross-frequency coupled components in the fit band. Note that this is also a valid simulation of an experiment when the entraining signal is known from other sources such as a cardiogram, a myogram, oscillatory signal from the transcranial alternating current stimulation, a visual or an auditory input etc. Recall Subsection 2.5 for details. For each pair

(8) $$\begin{array}{r} p:q=1:2,1:3,1:4,2:1,2:3,3:1,3:2,3:4,4:1,4:3 \end{array}$$

and each SNR = 1.0, 0.5, 0.1 we performed 100 simulations similar to the ones described in Subsection 2.7. The results are presented on Figure 2. We see a remarkably good performance for all SNRs and for all pairs *p*:*q* with the corresponding pattern divergence being < 0.05.

In this test we essentially eliminated all the errors which relate to the performance of SSD algorithm at the step of the initial extraction of the reference components. As we will show later, insufficiently clean extraction of SSD components can lead to reconstruction errors for the GCFD algorithm, compare Figures 2, **4**.

In general the results of this test demonstrate that the core optimization procedure performs well for all tested frequency ratios which are often met in E/MEG signal synchronization studies.

### 3.2. Simulations for GCFD algorithm

Simulations based on realistic head modeling showed that GCFD algorithm reliably recovers cross-frequency coupled components at different frequencies, relating to each other through rational numbers *p*:*q* up to, at least, *p, q* ≤ 4. This is a significant improvement over the baseline Cross-Frequency Decomposition (CFD) algorithm (Nikulin et al., 2012) which was only capable of dealing with the case *p* = 1 and *q* ≤ 3.

**Figure 3:**
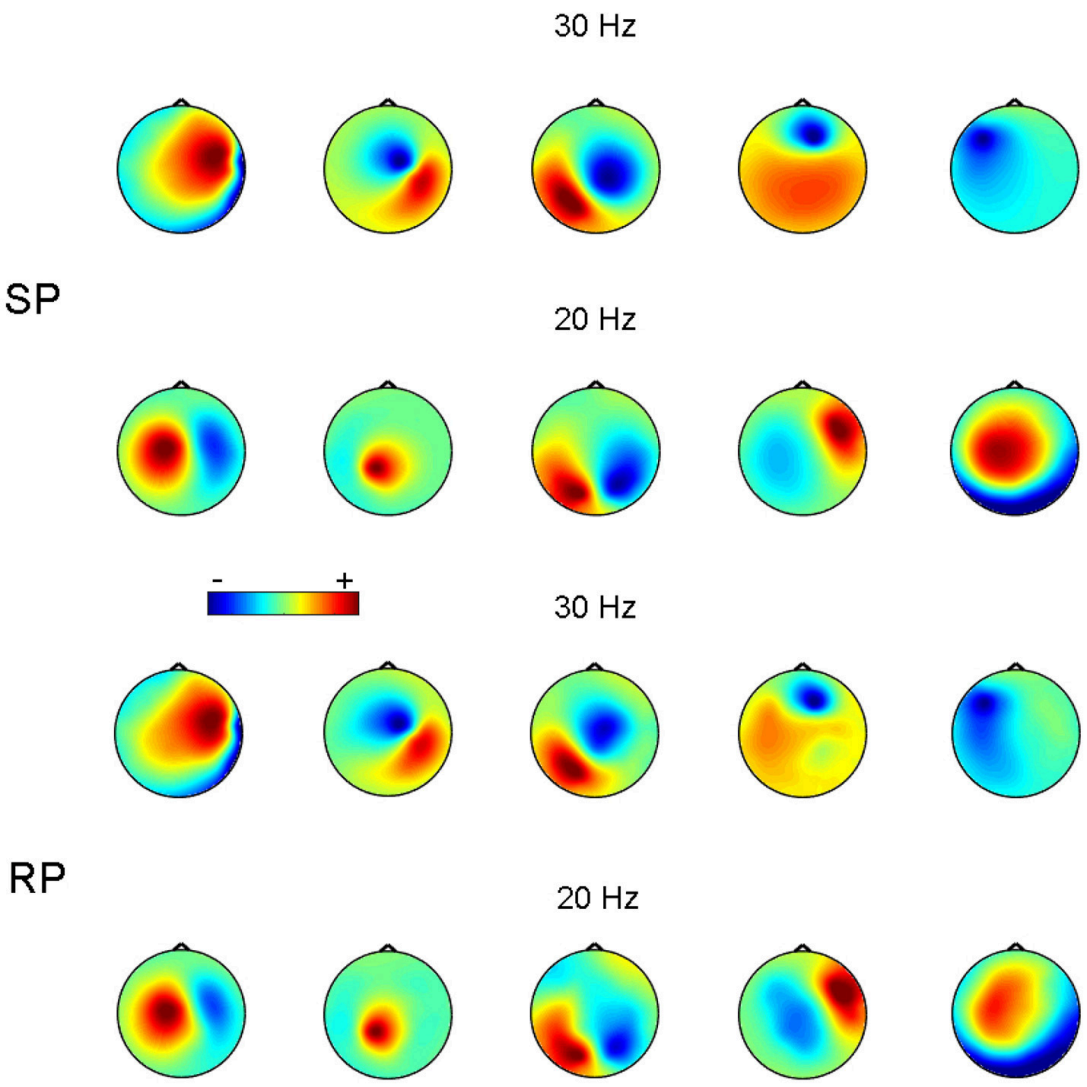
Source patterns (SP) and recovered patterns (RP) for 5 pairs of simulated synchronized sources at 20 and 30 Hz. SNR = 0.1. The color-scale is in arbitrary units.

Figure 3 shows an example of reconstruction of 5 simulated 30 Hz sources synchronized to 5 other 20 Hz sources. In this example, *p*:*q* = 3:2 and SNR = 0.1 for both 30 and 20 Hz frequency bands. Figure 3 demonstrates that the recovered topographies of 30 and 20 Hz sources were very similar to the simulated patterns.

**Figure 4:**
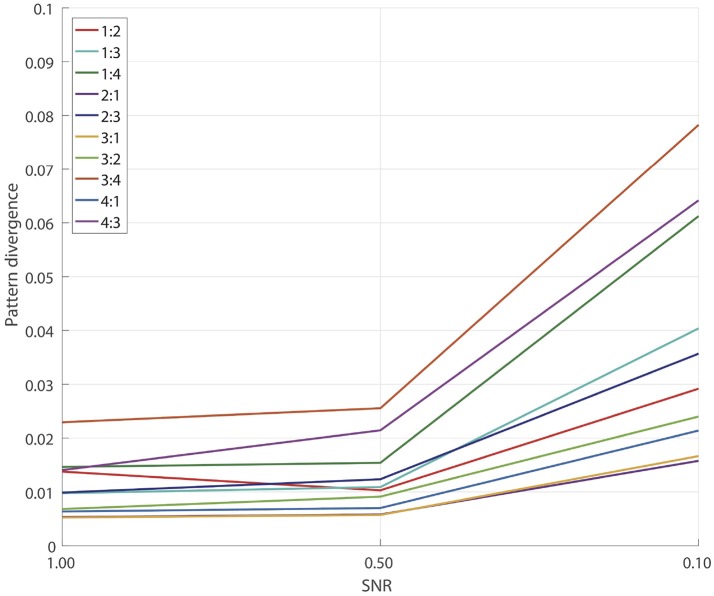
Pattern reconstruction accuracy for the whole GCFD.

To measure the overall pattern recovery quality of GCFD algorithm, we performed series of 100 simulations for each of frequency ratios (6) *p*:*q* = 1:2, 1:3, 1:4, 2:1, 2:3, 3:1, 3:2, 3:4, 4:1, 4:3 and SNRs = 1.0, 0.5, 0.1. The results are shown on Figure 4.

Naturally, as the SNR decreased, for each fixed ratio *p*:*q* we observed a gradual decrease in the pattern recovery accuracy. However, for all the frequency ratios even at SNRs ≥0.1 the median error was still very small, no greater than 0.08. The results of detached XPF test, see Subsection 3.1, indicate that a major source of the accuracy decay is due to insufficient performance of SSD.

Overall we concluded that for all tested frequencies and SNRs ≥0.1 the pattern recovery accuracy is sufficient for the analysis of synchronized sources in real E/MEG recordings.

When simulating uncoupled sources we observed that at SNR = 0.5 pattern divergence was on average 0.33 which was at least 15 times larger than the pattern divergence typical for coupled sources. Such values of pattern divergence indicate that the extracted topographies were very different from the topographies of the original uncoupled sources. This in turn indicates that in simulations where the sources are not coupled, GCFD is not able to recover simulated components.

### 3.3. Real EEG recordings

#### 3.3.1. Resting state

**Figure 5:**
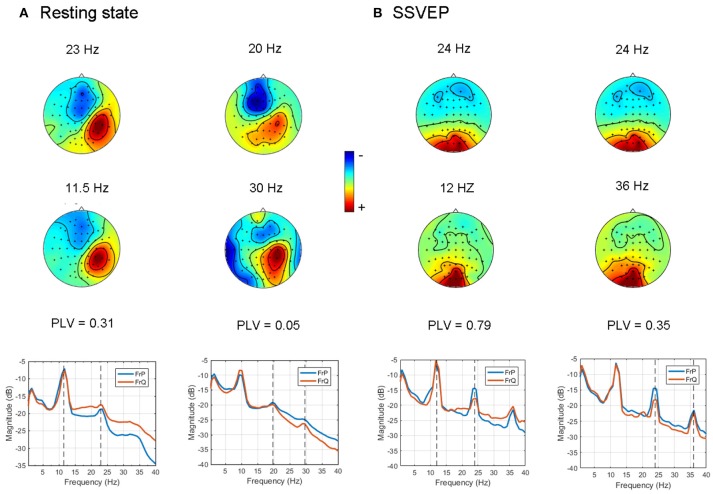
Examples of cross-frequency coupled synchronous oscillations detected with the GCFD algorithm for 2:1 and 2:3 search. **(A)** For resting state data. **(B)** For recordings with SSVEP 12 Hz.

**Figure 6:**
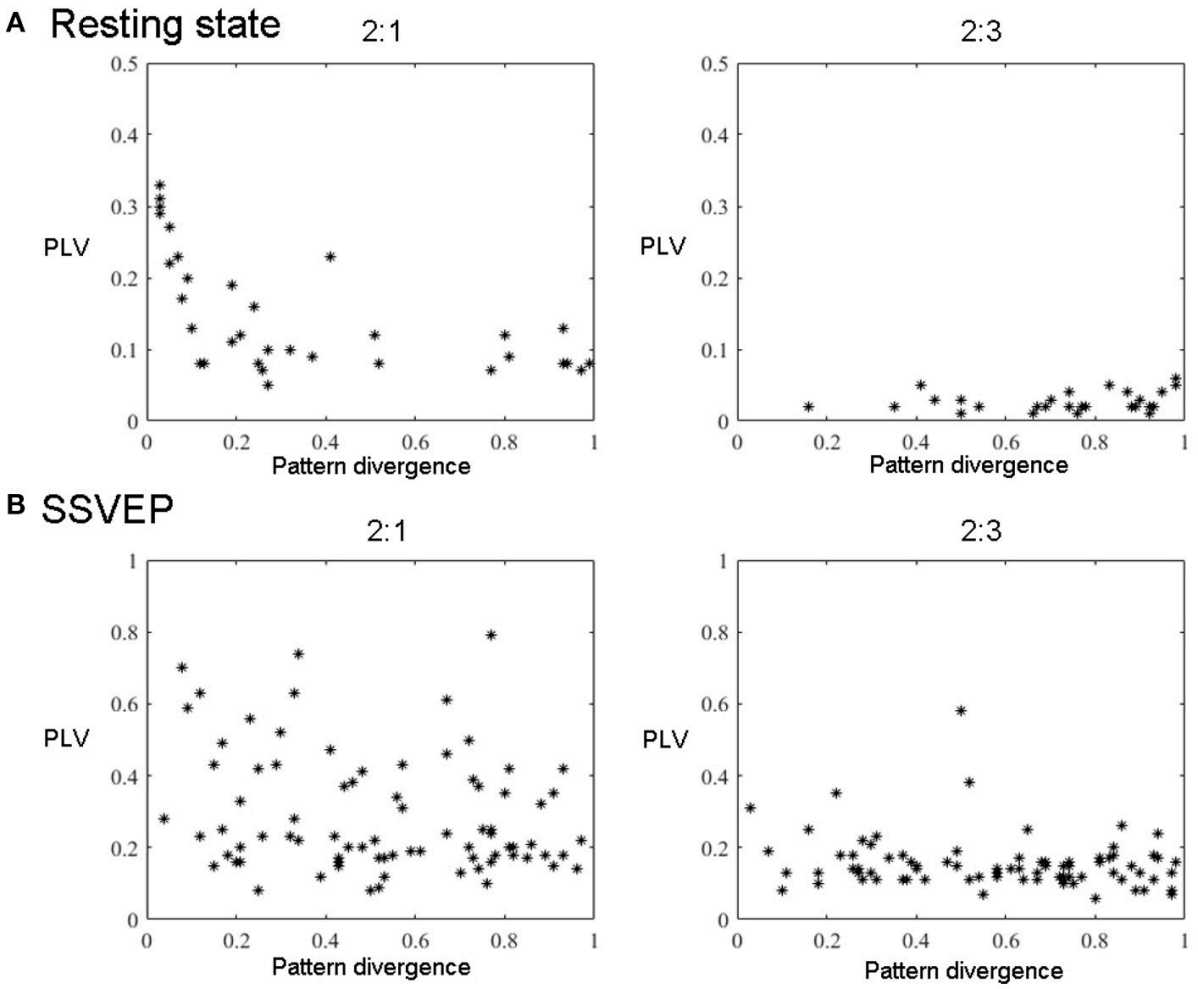
All components' pairs (2:1 and 2:3) from all subjects extracted with GCFD for 2:1 (left) and 2:3 (right) search. **(A)** For resting state data. **(B)** For recordings with SSVEP 12 Hz.

First we tested how the GCFD works for the resting state EEG recordings described in 2.9.1. We chose 8 subjects with the most pronounced power peaks in the alpha, beta and gamma frequency range and ran GCFD analysis to identify cross-frequency coupled synchronous sources. The base frequencies were taken from the alpha range 8–12 Hz. Figure 5A shows results for a typical subject for which we performed 2:1 and 2:3 searches with the base frequency equal to 9.9 Hz. Figure 6A represents all pairs of signals restored by the GCFD across all the participants.

For the frequency ratio 2:1 most (92%) computed phase locking values were statistically significant. However, the correlation between PLV and pattern divergence was not particularly strong (*R*^2^ = 0.49) thus indicating that only part of the data is likely to represent a coupling due to non-sinusoidal shape of neuronal oscillations.

For the frequency ratio 2:3 only 20% of the PLVs were statistically significant. We also analyzed the relationship between the strength of phase coupling and relevant pattern divergence. We observed a negative correlation for the case of 2:1 (*p*-value = 7·10^−6^) and no correlation for 2:3 (*p*-value = 0.11).

#### 3.3.2. Steady state visual evoked potentials

We also tested the GCFD on the Steady State Visual Evoked Potentials (SSVEP) signals described in 2.9.2. The goal was to demonstrate that our approach is able to find cross-frequency phase synchronized harmonics of SSVEP signals with frequencies relating to each other through a rational relationship *p*:*q* for *p, q* ≤ 4. We performed GCFD analysis across 24 subjects with flicker frequencies 5.45, 8.57, 12, and 15 Hz. We used the visual stimulation frequency as base frequency and fitted *p*:*q* relationship according to investigated harmonics of SSVEP signal. Figure 5B shows results from a typical subject with visual stimulation frequency 12 Hz, where 2:1 and 2:3 searches were performed. We observed pairs of signals with similar spatial patterns which suggest that the corresponding neuronal sources are both in the visual cortex. Moreover, we observed significantly high PLVs between components with different frequencies. The significance of the results was examined with permutation tests described earlier in Subsection 2.9.3. Permutation tests revealed that 44 and 28% of pairs were significant for 2:1 and 2:3 interactions, respectively. Figure 6B shows all pairs of signals found by GCFD in EEG recordings with stimulation frequency 12 Hz for 2:1 and 2:3 search. Pattern divergence was calculated according to the 7. It indicates similarity between the two topographies. No significant correlation between PLV and pattern divergence was observed for 2:1 (*p*-value = 0.10) and 2:3 (*p*-value = 0.13).

## 4. Discussion

We presented a new algorithm for the detection and extraction of cross-frequency phase-to-phase synchronized neuronal components. Generalized Cross-Frequency Decomposition is able to reconstruct both the time courses of synchronized neuronal components and corresponding spatial filters and patterns.

We showed that the GCFD was capable of detecting synchrony between frequencies related by a rational relationship p:q, for *p, q* ≤ 4. The new method extends the previous state of the art, Cross-Frequency Decomposition (CFD) (Nikulin et al., 2012) to a more general range of frequency pairs. However, for *p, q* > 4 a detection of cross-frequency synchronization is difficult because the phase-locking region is very narrow and a possible synchrony is likely to be obscured by noise. This is a general problem of cross-frequency interactions' detection which is not specific to the GCFD algorithm (Pikovsky et al., 2001).

### 4.1. Limitations of previous methods

The most common approach is to calculate cross-frequency synchronization in sensor space (Schanze and Eckhorn, 1997; Tass et al., 1998; Carlqvist et al., 2005; Palva et al., 2005; Nikulin and Brismar, 2006; Sauseng et al., 2008; Darvas et al., 2009; Siebenhühner et al., 2016). One of the pitfalls of the analysis in sensor space is that the source topographies cannot be identified due to a mixing problem related to volume conduction. Another approach (Tass et al., 2003) is based on the inverse modeling of source signals which are then pair-wise checked for the presence of cross-frequency synchrony by computing Phase Locking Values across all the brain voxel pairs. This approach requires extensive corrections for type I statistical errors. Furthermore, these comparisons generate great complexity of the neuronal relationships which, however, does not necessary relate to the true neuronal complexity. In case of the inverse modeling it is also important to pay attention to the general ambiguity of the inverse reconstruction.

### 4.2. Advantages and limitations of GCFD

Numerical simulations showed that GCFD can recover interacting sources even when they are masked by a very strong noise (SNR = 0.1), see Figure 4. The extraction of the non-linearly interacting components was remarkably good showing only a small error (< 0.08) in recovered patterns even at a very challenging situation of SNR = 0.1. In this study we use SSD that is one of the best methods for the extraction of signals with low signal-to-noise ratio (Nikulin et al., 2011). The GCFD depends on the performance of SSD, in the way that SSD extraction errors for reference signals might influence final pattern reconstruction quality, see Subsections 2.3.2, 3.1. The GCFD has also performance asymmetry with respect to *p* and *q* swap due to its dependence on initial SSD. We showed in simulations that the detached XPF, which uses already available reference signals, performs significantly better compared to the whole GCFD, see the section 3. However, GCFD is not limited to the use of SSD to obtain reference signals. Here one can utilize components extracted with other decomposition approach such as ICA or directly available signals such as a cardiogram, myogram, visual or auditory signal etc. This can be particularly useful when analyzing cross-frequency corticomuscular interactions where reference signals are obtained with the surface EMG. In fact in a recent review, an importance of cross-frequency interactions was emphasized for a better understanding of cortical-spinal motor control (Yang et al., 2017). We believe that GCFD method can be a valuable approach in this regard.

### 4.3. Genuine and spurious cross-frequency interactions

While looking for cross-frequency synchronization, there is always a possibility to detect CFS not due to genuine neuronal interactions, but also due to non-sinusoidal shape of oscillations (Gaarder and Speck, 1967; Jürgens et al., 1995; Nikulin and Brismar, 2006). This is due to the fact that non-sinusoidal waveforms represent a sum of sine waves at the fundamental and harmonic frequencies (which are integer-multiples of the base frequency), and these sine waves would then demonstrate cross-frequency synchronization. For example, (Nikulin and Brismar, 2006) have shown in a numerical experiment that when the EEG signal is non-sinusoidal, it exhibits spurious alpha-beta phase coupling.

In order to control for this side-effect, we calculated the pattern divergence between the spatial patterns of the reference signal and the synchronized rhythmic components. If the patterns were similar we considered them to be harmonics. For 2:1 case we observed many similar patterns with high PLV. This was not the case for 2:3 coupling where we observed a smaller number of similar patterns and the average PLV was lower.

We showed that GCFD can be applied to find phase coupling in cases when we use pre-selected peaks on the basis of the spectra as shown for resting state Figure 5A, and also for cases when a priori information is known about the reference spectra, which is the case for SSVEP experiment, Figure 5B. All pairs found by GCFD are presented in Figure 6. In EEG experiments we did not find particularly strong correlation between pattern similarity and the strength of cross-frequency phase synchrony. This in turn indicates that the extracted cross-frequency interactions are unlikely to be due to the presence of harmonics since in that case a correlation between the pattern similarity and PLV values would be very strong. Moreover, a sensitivity of GCFD both to genuine and “harmonic-like” cross-frequency interactions has further advantages, since it might allow a more precise extraction of non-sinusoidal neuronal oscillations important for Brain-Computer Interface systems based on mu-rhythm known to have second and even third harmonics. The presence of cross-frequency interactions in the resting state dynamics, as we observed in the present study, is particularly interesting case as this might indicate computational readiness of the neuronal networks to be engaged in the processing of information distributed across different cortical areas producing oscillatory activity at different frequencies.

As the direction for the future research, it would be interesting to apply GCFD to investigate the role of cross-frequency phase synchrony between different networks, demonstrating strong within-frequency coupling, in a variety of cognitive tasks suggested to engage such integration, e.g., in visual working memory (Siebenhühner et al., 2016). Our work demonstrates that the GCFD algorithm can be readily utilized for the investigation of such complex cross-frequency interactions.

## Ethics statement

This study was carried out in accordance with the recommendations of HSE Ethics Committee with written informed consent from all subjects. All subjects gave written informed consent in accordance with the Declaration of Helsinki. The protocol was approved by the HSE Ethics Committee.

## Author contributions

DV designed study, performed research, wrote the paper. ID performed research, wrote the paper. AM performed research, wrote the paper. BG and VN designed study, wrote the paper.

### Conflict of interest statement

The authors declare that the research was conducted in the absence of any commercial or financial relationships that could be construed as a potential conflict of interest.
